# Full-Endoscopic Interlaminar Approach for a Posteriorly Migrated Lumbar Disc Sequestration in a Patient Presenting with Cauda Equina Syndrome and Atypical Magnetic Resonance Imaging Findings: Case Report

**DOI:** 10.3390/reports9040361

**Published:** 2026-10-19

**Authors:** Marios Theologou, Kassiani Boulogeorgou, Nikolaos Syrmos, Nicoleta Pastelli, Panagiotis Kyriakongonas

**Affiliations:** 1 Department of Neurosurgery, General Hospital of Thessaloniki Georgios Papanikolaou, 57013 Thessaloniki, Greece; 2 First Department of Neurosurgery, IASO General Clinic, 15123 Athens, Greece; 3 Department of Pathology, General Hospital of Thessaloniki Georgios Papanikolaou, 57013 Thessaloniki, Greece; 4 School of Medicine, Aristotle University of Thessaloniki, 54124 Thessaloniki, Greece; 5 Department of Neurosurgery, 401 General Military Hospital of Athens, 11525 Athens, Greece

**Keywords:** lumbar disc herniation, full-endoscopic discectomy, sequestrectomy, epidural, cauda equina syndrome

## Abstract

**Background and Clinical Significance**: Posterior epidural migration of a sequestered disc fragment is a very rare entity that may present significant diagnostic challenges, due to radiological characteristics that may mimic other lesions; **Case Presentation**: A young adult male presented with acute deteriorating low back pain, paraparesis, and urinary incontinence. Magnetic Resonance Imaging (MRI) revealed a massive posterior intraspinal mass causing critical canal stenosis. Although the dorsal location and severe mass effect initially suggested a neoplasm, the complete absence of contrast enhancement strongly pointed toward an avascular lesion. Given the patient’s rapid neurological decline, an emergency full-endoscopic left-sided L_4_-L_5_ interlaminar approach was employed, and a massive free disc fragment was removed. The patient achieved complete motor and sphincter recovery within a month, with mild paresthesia resolving by six months; **Conclusions:** Posterior epidural migration of a sequestered disc fragment is a rare entity that should be included in the differential diagnosis of posterior intraspinal masses with atypical MRI characteristics. The full-endoscopic interlaminar approach is a viable minimally invasive surgical option.

## 1. Introduction and Clinical Significance

Lumbar disc herniations present the most common pathology encountered in spine surgery. Anatomically they can be classified into protrusions (disc bulging with an intact annulus), extrusions (disrupted annulus with disc material in the epidural space) and sequestrations (free fragments completely detached from the disc space) [1]. Sequestered disc fragments typically migrate along the cranial, caudal and lateral planes within the anterior epidural space [2,3]. Dorsal migration is an extremely rare finding [4]. Due to its rarity, this condition can be misidentified as other pathologies occupying the posterior epidural space, leading to diagnostic and treatment pitfalls (e.g., delayed management; inappropriate surgical planning) [5]. We report a rare case of a massive, dorsally migrated disc herniation presenting with acute cauda equina syndrome, paraparesis and atypical neuroimaging findings. We discuss the pathophysiological mechanisms leading to posterior migration, the diagnostic and treatment challenges associated with the atypical presentation and present the endoscopic surgical anatomy of the interlaminar full-endoscopic lumbar spine decompression.

## 2. Case Report

### 2.1. Clinical Presentation

A 39-year-old Caucasian male presented to the emergency department with a two-week history of progressively worsening low back pain, which acutely deteriorated over a 48-h period into a progressive lower extremity weakness. He denied any history of recent trauma, heavy lifting or intense physical activity. Prior medical history was unremarkable.

On admission, neurological examination revealed bilateral motor deficits. Motor strength was graded using the Medical Research Council (MRC) scale.

- Right lower extremity: tibialis anterior (3/5); extensor hallucis longus (3/5), peroneus muscles (3/5), gastrocnemius (3/5), flexor hallucis longus (3/5)
- Left lower extremity: tibialis anterior (2/5); extensor hallucis longus (2/5), peroneus muscles (3/5), gastrocnemius (3/5), flexor hallucis longus (3/5)

Deep tendon reflexes were slight (1+) and symmetric. Pathologic reflexes were absent. The patient reported severe pain, noting a Visual Analog Scale (VAS) score of 7/10 for back pain and 9/10 for leg pain. Functional impairment was graded severe, with an Oswestry Disability Index (ODI) score of 74%. A bilateral hypesthesia was also recorded from L_4_ and below. While undergoing further evaluation he developed partial urinary incontinence—signaling the evolution of Cauda Equina Syndrome Incomplete (CESI).

### 2.2. Radiological Evaluation

**Figure 1:**
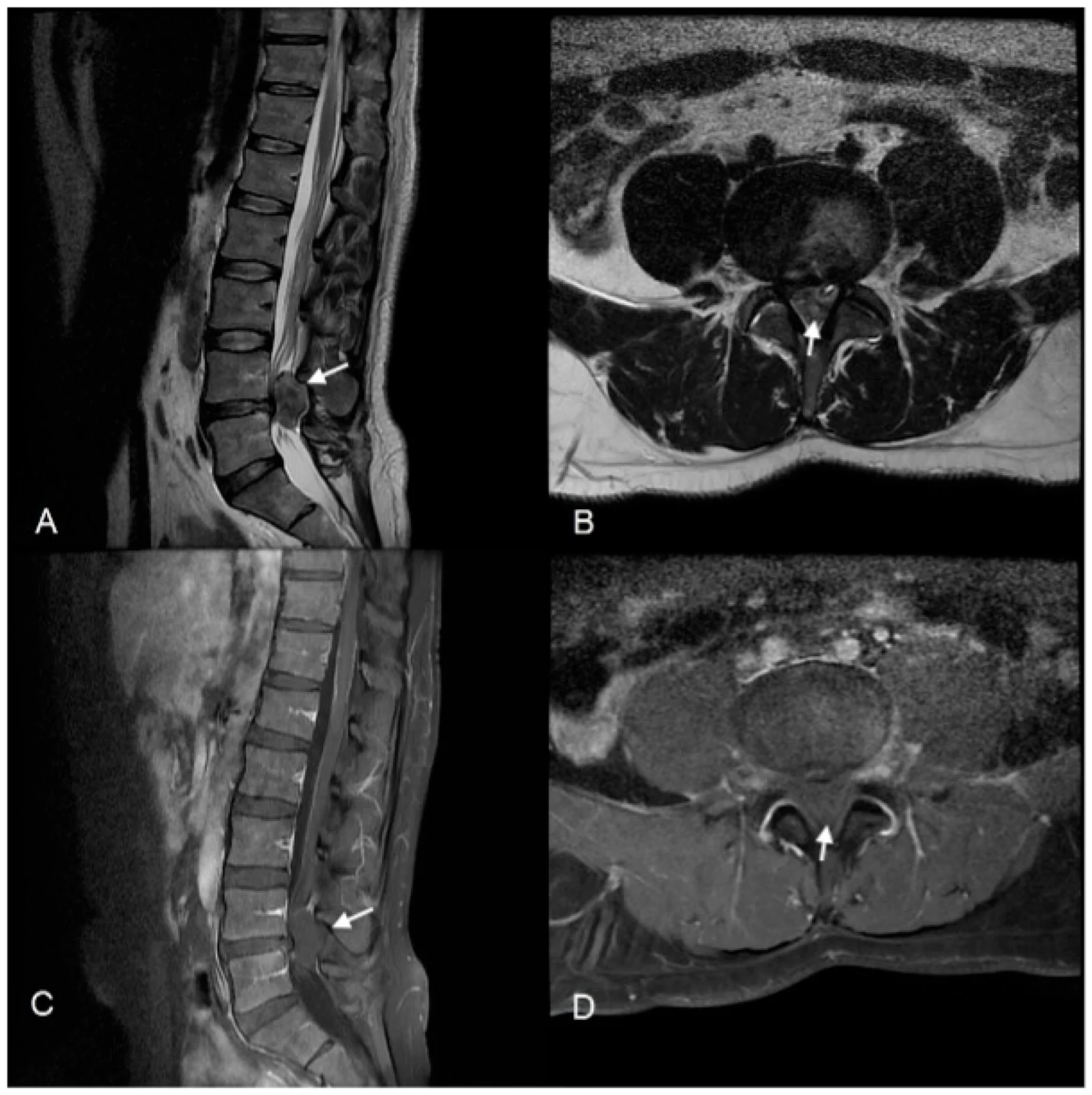
(**A**) Sagittal T2-weighted and (**B**) axial T2-weighted image revealing the presence of an L_4_-L_5_ tumor-like formation (arrow) emanating posteriorly, almost entirely occluding the spinal canal, causing an anterior and lateral sac migration, with severe compressive effect. (**C**) Sagittal T1-weighted and (**D**) axial T1-weighted image post-gadolinium application showing no enhancement of the lesion.

An urgent magnetic resonance imaging (MRI) study of the lumbar spine was performed within 1 h of CESI evolution (Figure 1).

### 2.3. Surgical Management

**Figure 2:**
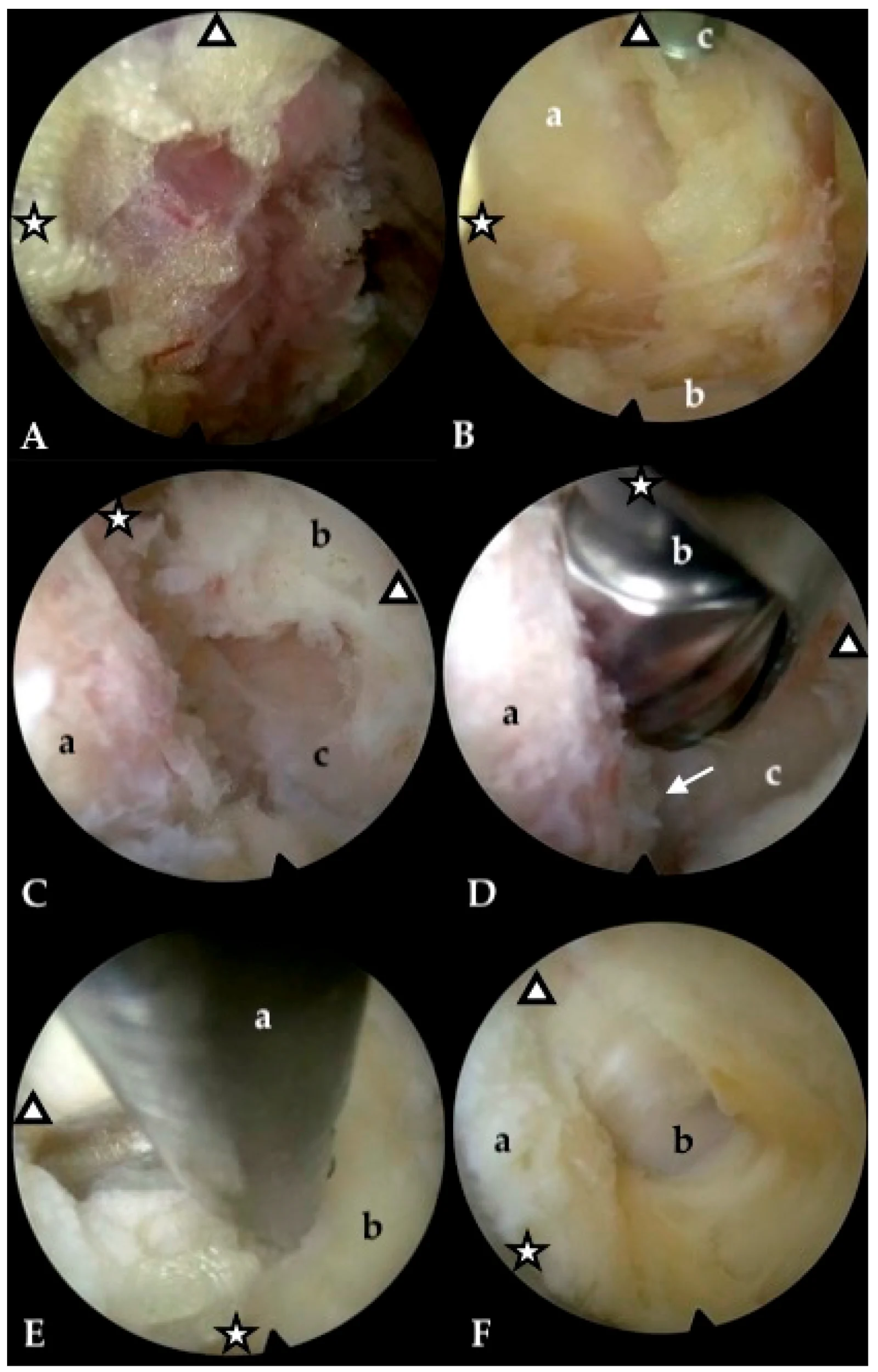
Full endoscopic surgical anatomy. (Star points to the cranial triangle to medial) (**A**) Initial visualization upon introduction of endoscope: muscle, adipose and connective tissue. (**B**) Tissue dissection: a—ligamentum flavum; b—facet joint capsula; c—RF cautery probe. (**C**) Localizing the tip of the descending part of the facet: a—tip of the descending part of the facet; b—ligamentum flavum deep attachment to ascending part of the facet; c—ascending part of the facet. (**D**) Bone resection: a—tip of the descending part of the facet. Arrow—synovial/joint line; b—cutting burr with side protection; c—ascending part of the facet. (**E**) Opening the ligamentum flavum: a—scissors applied cutting the ligamentum flavum as medial as possible in a forehand position and with the optic rotated in a manner securing visualization of the tip of the instrument; b—ligamentum flavum. (**F**) a—ligamentum flavum (stretched by the working channel); b—discoloration corresponding to the deep layer of the ligamentum flavum.

**Figure 3:**
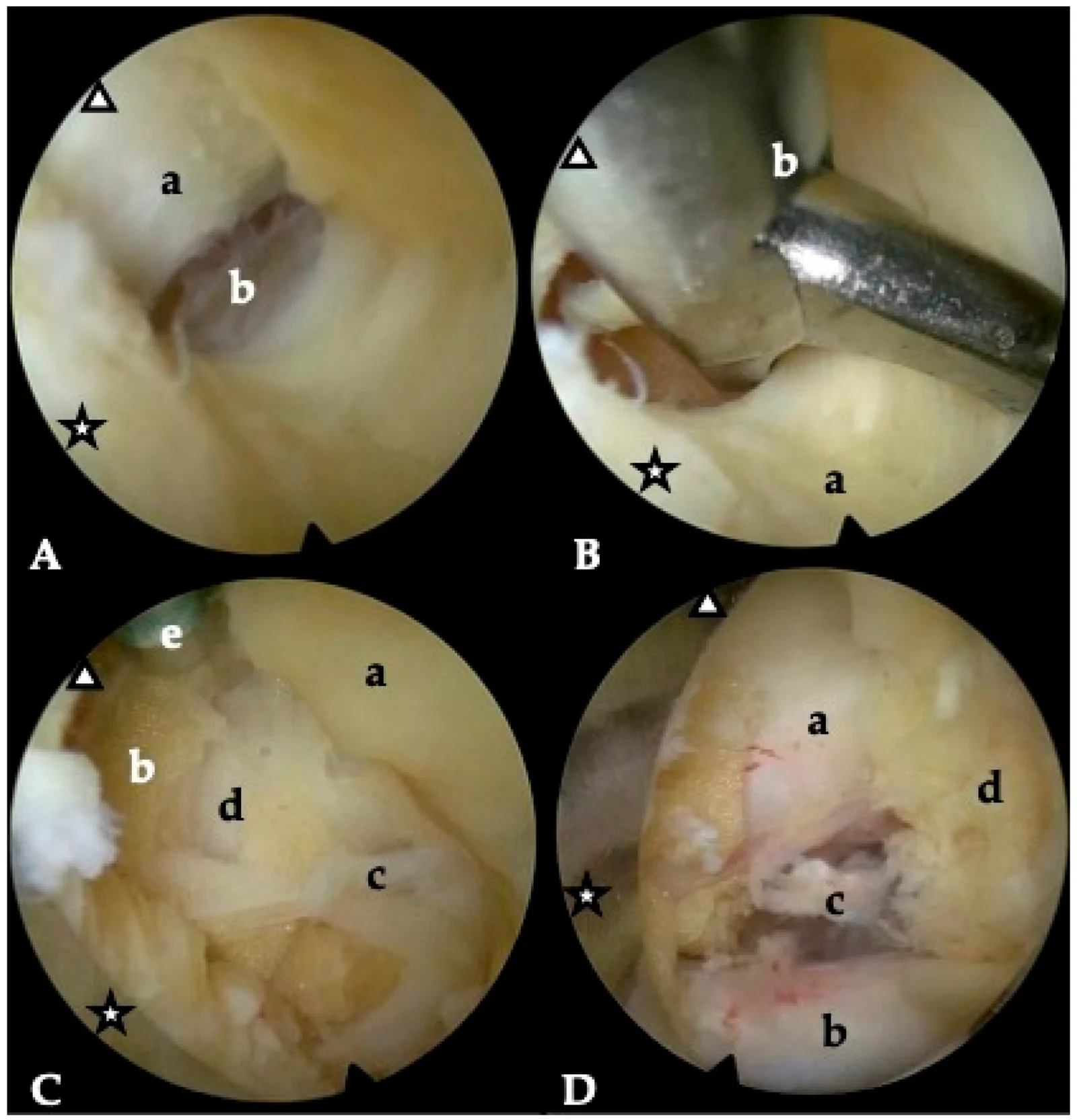
Full endoscopic surgical anatomy presentation. (Star points to cranial triangle to medial) (**A**) a—ligamentum flavum; b—flavum dehiscence. (**B**) a—ligamentum flavum; b—scissors used in backhand position from medial to lateral. (**C**) a—ligamentum flavum; b—adipose tissue; c—vascular structures; d—lesion creating an outward compression of the peridural membrane; e—tip of RF cautery probe. (**D**) Post lesion resection; a—dura; b—traversing nerve root; c—axilla; d—epidural adipose tissue.

**Figure 4:**
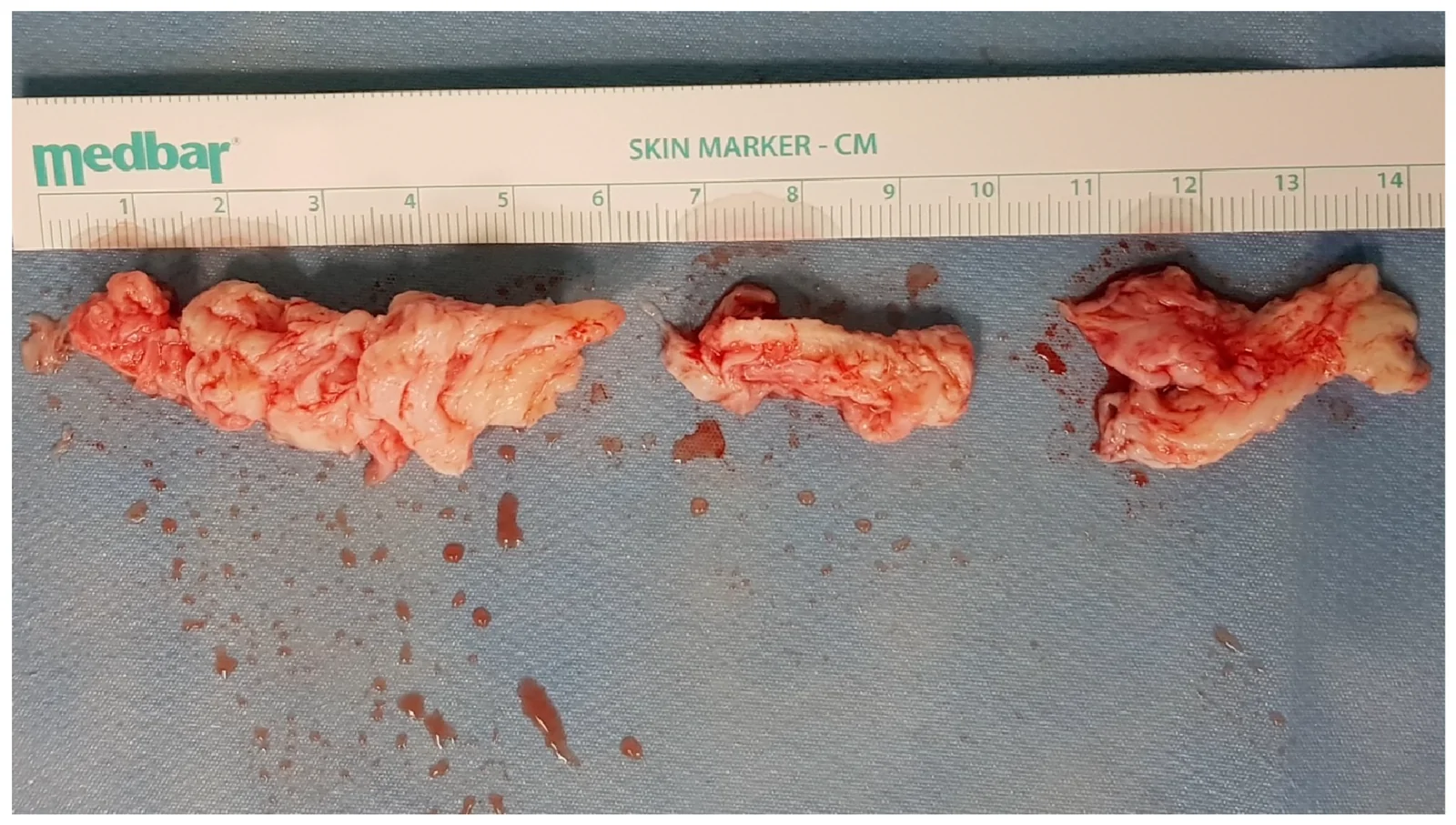
Extracted disc material (metric scale in centimeters provided).

**Figure 5:**
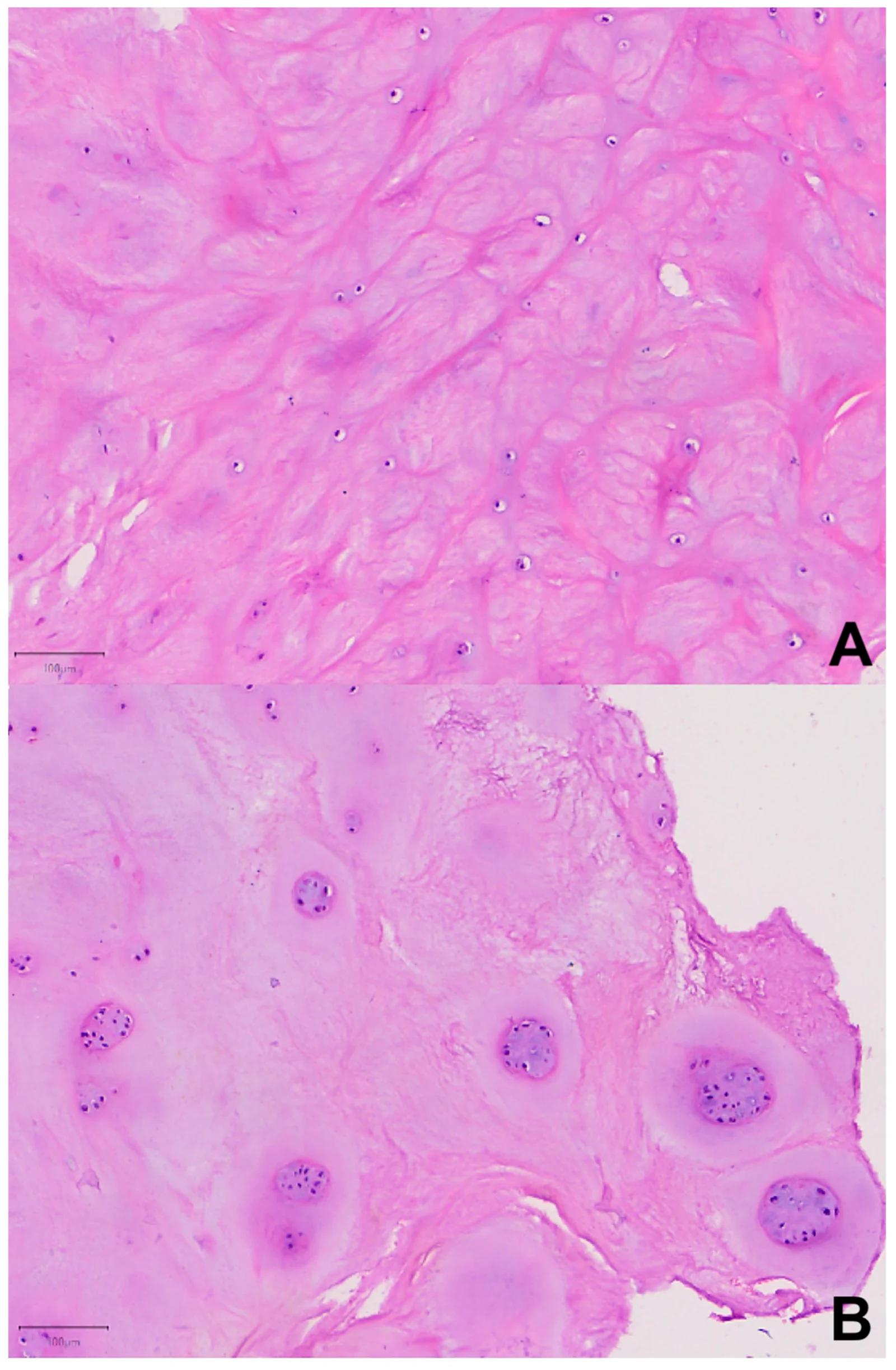
(100 μm scale provided) Intervertebral disc: hematoxylin-and-eosin-stained sections show fragments of loose fibromyxoid matrix (nucleus pulposus), rich in stellate, fusiform cells (**A**) and chondrocytes organized in small groups (**B**).

Given the patient’s acute neurological deterioration, emergency surgical intervention was undertaken (7 h post MRI evaluation) employing a left sided L_4_-L_5_ full endoscopic interlaminar approach (Figure 2 and Figure 3). Under general anesthesia, the patient was positioned prone in an anti-lordotic stance. Fluoroscopic guidance was used to place a 1 cm incision on the radiographic projection of the interlaminar window’s midpoint, also stabbing the thoracolumbar fascia. The dilatator was introduced followed by the positioning of an Ø 8 mm working channel and an oval 25° 6.9 mm discoscope (VERTEBRIS Lumbar^®^, RIWOspine GmbH, Knittlingen, Germany), connected to a high-definition camera system. Continuous irrigation was achieved using a fluid control pump, set to spine-mode to regulate inflow pressure, preventing intraspinal and secondary intracranial pressure increase. A 4-MHz radiofrequency surgical cautery system was used for dissection and hemostasis. The anatomical structures were dissected (Figure 2A,B). An oval cutting burr with side protection was used to undercut the facet and cranial lamina, facilitating sufficient space for unobstructed movement of the working channel (Figure 2C,D). The ligamentum flavum was incised (Figure 2E,F and Figure 3A,B) employing scissors, gaining access to the spinal canal. The lesion was identified immediately below the flavum surrounded by the adipose tissue and vascular structures of the peridural membrane (Figure 3C). It was extirpated decompressing the neural structures, which were then mobilized for further inspection (Figure 3D). The lesion was macroscopically attributed to a disc sequester (Figure 4), something that was also histopathologically confirmed (Figure 5). Prior to closure a mixture of 125 mg of methylprednisolone, 4 mL of 2 mg/mL ropivacaine and 4 mL of water for injection was locally applied through the working channel. Operative time was approximately 1 h, and blood loss was approximately 15 mL (estimated by hematocrit method—This is based on the ability of red blood cels (RBC) to maintain their morphology in 0.9% saline for a significant time. The rinse solution was collected meticulously from a 3M™ Steri-Drape Pouch into a canister after being passed through a CellTricks™ 20 μm gravity flow filter to clear from tissue debris. The hematocrit and volume of the solution were measured. The volume of lost blood was calculated ($\mathrm{Vbloodloss}\;=\;\frac{\mathrm{Hct}\;\mathrm{rinse}\;\mathrm{solution}}{\mathrm{Hct}\;\mathrm{normal}\;\mathrm{blood}}\;\times \;\mathrm{Vsolution})$ [6,7]). The surgical procedure was completed uneventfully.

### 2.4. Postoperative Course

The patient manifested a rapid recovery of motor function

• Right lower extremity: tibialis anterior (4/5); extensor hallucis longus (4/5), peroneus muscles (4/5), gastrocnemius (4/5), flexor hallucis longus (3/5)
• Left lower extremity: tibialis anterior (4/5); extensor hallucis longus (3/5), peroneus muscles (3/5), gastrocnemius (4/5), flexor hallucis longus (4/5)

With complete resolution of radicular pain (VAS leg 1/10) and only low-back discomfort (VAS back 2/10). He was discharged after 24 h, being transferred to a rehabilitation center. At the 2-week follow-up he presented with a complete resolution of all motor deficits and sphincteric dysfunctions. He also denied any leg or back pain. Paresthesia gradually improved a month post-surgery, with mild discomfort located solely on the dorsal region of the left foot persisting for a 6-month period. No postoperative imaging was performed.

## 3. Discussion

Sequestered disc fragments are frequently encountered in routine spine surgery, representing approximately 1.7–3% of all lumbar disc herniations [8,9]. Among these, the posterior epidural migration represents an extremely rare phenomenon, with the existing literature being limited to sporadic case reports and case series [4].

The exact pathophysiological mechanisms leading to this atypical migration pattern have not been completely understood yet. Under physiological conditions, the Posterior Longitudinal Ligament (PLL), the peridural and lateral membranes act as rigid barriers confining the herniated disc material within the anterior epidural space. Furthermore, the nerve root itself, the epidural fat and the extensive network of the epidural vascular structures provide additional mechanical resistance [10]. Consequently, for a free disc fragment to migrate behind the dural sac, it must breach or bypass these protective structural boundaries.

Factors predisposing the dorsal migration can be broadly categorized into extrinsic (lifestyle/occupational) and intrinsic (anatomical/biomechanical). Extrinsic factors include heavy labor, traction and spinal manipulation [11]. Intrinsic factors include conditions of segmental hypermobility, an increased sagittal and/or axial spinal canal diameter (providing a path of less resistance around the neural structures), the degree of the concavity of the posterior part of the vertebral body and the spatial relationship between the nerve root, the dura and the annular tear [11,12].

**Figure 6:**
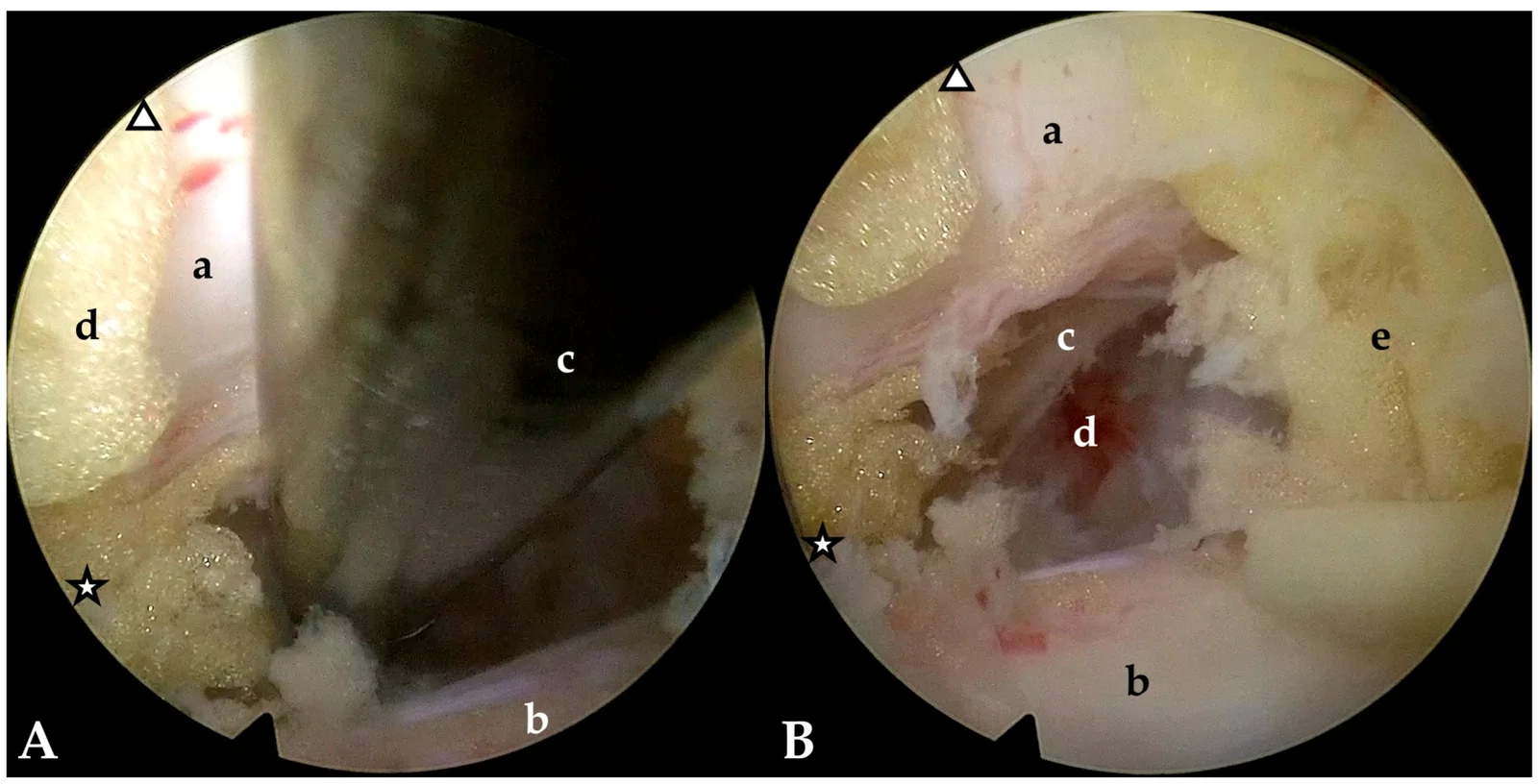
Full endoscopic surgical anatomy presentation. (Star points to cranial triangle to medial). (**A**) a—dura; b—medial root border; c—rongeur used to localize primary PLL tear by palpation; d—peridural adipose tissue. (**B**) a—dura; b—nerve root; c—PLL; d—PLL primary tear; e—peridural membrane/adipose tissue remnants.

In the presented case, the lesion was identified immediately beneath the ligamentum flavum, with a thin layer of adipose tissue partially covering it, alongside some stretched, probably thrombosed vascular structures (Figure 3C). The primary PLL tear was identified at the level of the caudal vertebral endplate, immediately adjacent to the nerve root axilla (Figure 6). This specific location likely facilitated the fragment’s posterolateral migration into the dorsal space.

Epidemiological data confirm that posterior epidural migration occurs predominantly in males and exhibits a profound anatomical predilection for mid-to-lower lumbar spine, with the vast majority of cases involving the L_4_-L_5_ level (35%), followed by the L_3_-L_4_ level (32%) [13]. According to some authors this anatomical distribution is theoretically associated with the more horizontal inclination of these disc spaces relative to the lumbosacral junction [14], combined with the larger canal diameter (compared to upper levels), leading to an increased incidence of failure to constrain the free fragment in the anterior epidural space. From a biomechanical perspective, the L_3_ and L_4_ vertebrae actively endure substantial loads in both sitting and standing postures, often exceeding more than twice the total body weight [15]. In theory this massive load can generate a significantly high intradiscal pressure triggering an inside-out burst tearing the annulus fibrosus apart, expelling a large free fragment with sufficient kinetic energy to pierce and displace the anatomical boundaries in its way to the posterior compartment. The presented case supports the epidemiological data presenting at the L_4–5_ level.

Differentiating a posteriorly migrated intervertebral disc sequestration from other space-occupying lesions within the dorsal part of the spinal canal—including cysts, hematomas, abscesses and neoplasms—may present a diagnostic challenge in some cases [10,16] (Table 1). Contrast-enhanced MRI remains the diagnostic tool of choice, typically presenting T1-weighted isointensity, T2-weighted hyperintensity relative to the parent disc and a characteristic peripheral ring-enhancement following gadolinium administration [12]. However, the presented case defied these classic imaging patterns. While the dorsal location of the mass, alongside the imaging characteristics of T1 and T2 weighted sequences, initially raised the suspicion of an intraspinal neoplasm, the paradoxical absence of contrast enhancement pointed toward an avascular structure. The absence of peripheral ring enhancement, which typically reflects an inflammatory response characterized by macrophage infiltration, neovascularization and peripheral granulation tissue formation, can likely be attributed to the early timing of diagnosis and treatment. The rapid onset and hyperacute progression of neurological deficits strongly directed the diagnostic impression toward a massive disc sequestration.

Patients with posterior epidural migration of a disc fragment (PEMDF) frequently present with acute severe neurological symptoms, including Cauda Equina Syndrome (CES) and severe motor deficits, due to the critical mass effect within the posterior compartment of the spinal canal [16]. In contrast, patients with spinal neoplasms typically present with a more insidious, subacute or chronic deterioration.

Patients presenting with lumbar disc herniations resulting in mild pain and no neurological findings may be treated conservatively, as rates of spontaneous regression may be as high as 96%, especially in cases of disc extrusions and sequestrations [22,23].

Emergency surgery is the treatment of choice for those presenting with neurological deficits. Various decompressive approaches (laminectomy, hemilaminectomy) may be opted employing minimally invasive techniques (microscopically assisted, endoscopically assisted or full-endoscopic). To our knowledge, the vast majority of published cases have been treated with microscopically assisted decompression techniques, with the application of Full-Endoscopic Spine Surgery (FESS) for dorsally migrated sequestrations remaining exceptionally sparse [24]. The adoption of FESS approaches presents a growing trend especially in the lumbar and cervical regions; driven by potential advantages [25,26,27]. These can be divided into technical (superior operating ergonomics, high-quality visualization that can reveal otherwise obscured anatomical and pathological structures by placing the optic directly adjacent to them (e.g., beneath the neural structure or inside the foramen) and clinical outcome advantages (diminishing surgical site infections rates, less intraoperative blood loss, better preservation of the paraspinal musculature, ligaments and bony structures, reduction of local and systemic inflammation, faster functional recovery and a superior cosmetic result) [27,28,29]. Notably, while literature demonstrates a statistically significant reduction in surgical site infections with FESS compared to microdiscectomy, other clinical parameters show favorable trends without reaching statistical significance.

Certain technical limitations must be acknowledged, however, when using FESS. Although modern systems feature high-definition optics (4K), visual feedback remains two-dimensional (2D). While three-dimensional (3D) reconstruction technologies are available, their clinical adoption remain limited, and their added value has yet to be proven. This lack of stereoscopic depth perception can be challenging, particularly for less experienced surgeons, and may be one of the factors responsible for the observed flat learning curve. Furthermore, due to the restricted diameter of the discoscope’s working channel and sleeve, a large fragment can completely occlude the endoscopic visual field, requiring securing the sleeve in a fixed position and withdrawing the fragment alongside the optical system, a maneuver that briefly interrupts the direct visualization of the spinal canal (Supplementary Material—video presentation).

A comparative analysis of published microscopic versus full-endoscopic cases for dorsally migrated sequestrations was not feasible due to lack of available data regarding the latter.

**Figure 7:**
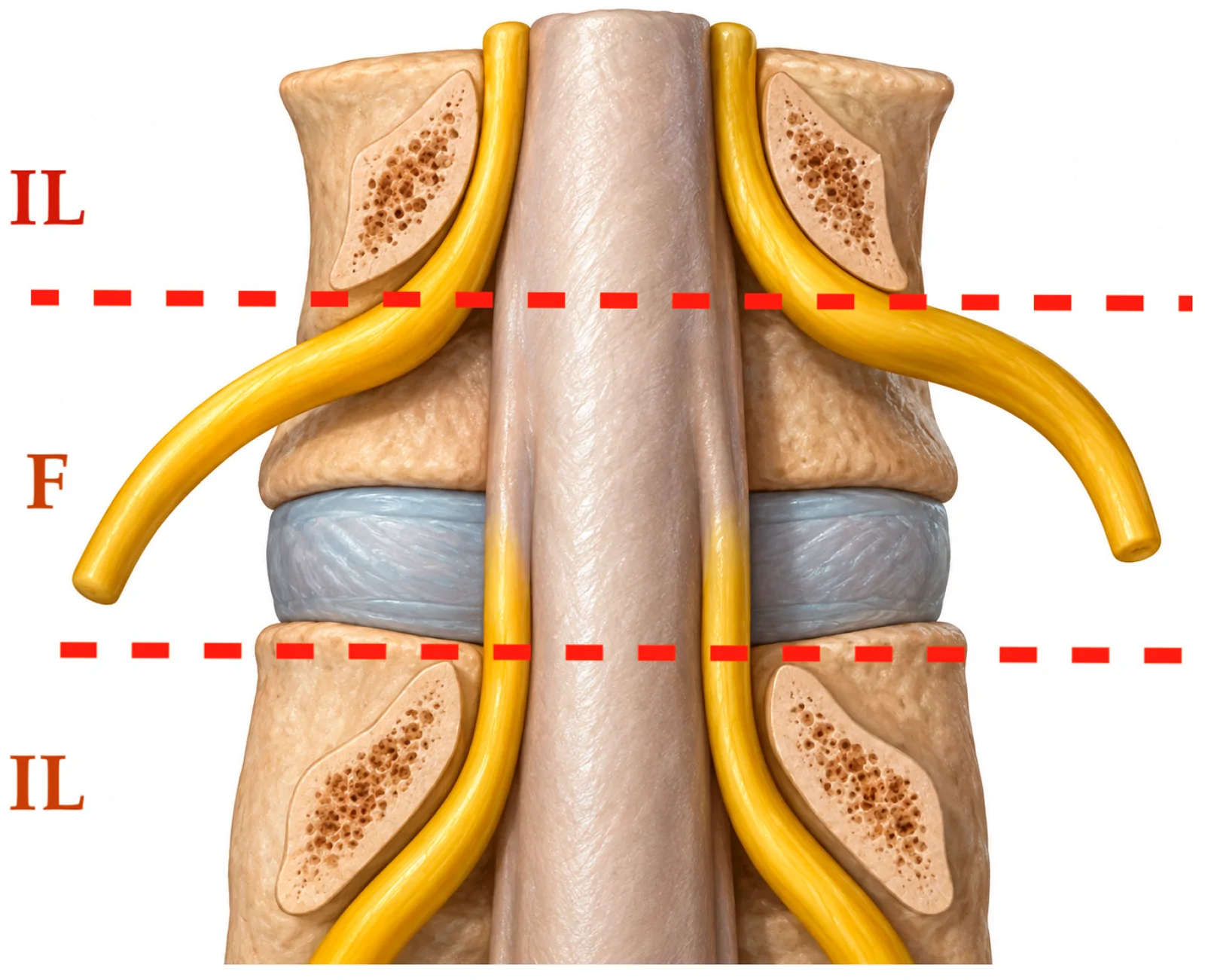
Protocol for choosing between interlaminar and transforaminal approach for disc herniations (does not apply for posterior migration or cases with lateral recess and central canal stenosis, which are always treated with an interlaminar approach). IL—interlaminar approach mandatory, F—foraminal approach feasible for central herniation—transforaminal approach; foraminal herniation—intraforaminal approach; lateral/extraforaminal—extraforaminal approach.

Taking into consideration the highlighted advantages and limitations, the surgical team opted for the FESS. We should highlight that our surgical team chooses FESS for all single level spinal canal stenoses (unilateral or bilateral) and disc herniations, choosing for the latter between interlaminar and transforaminal approach according to the location of the pathology (Figure 7). Microscopic surgery is reserved for multilevel spinal canal stenosis (>2 levels), revision surgery and in case of an extensive accidental dural tear requiring suturing.

Patient recovery typically requires a significant period, often measured in months. Surgical timing remains a paramount prognostic factor, with intervention within 48 h of CES onset offering significantly improved outcomes [30,31]. The excellent and rapid clinical recovery observed in the presented case, may be potentially attributed to the early surgical decompression (<12 h post CESI manifestation), the incomplete presentation of CES and the patient’s young age.

The local epidural application of a steroid and local anesthetic combination was used in accordance with institutional protocol to alleviate early postoperative axial and radicular pain, something supported by the existing literature [32,33].

## 4. Conclusions

Posterior epidural migration of a sequestered disc fragment is an exceedingly rare manifestation that should be considered in the differential diagnosis of posterior epidural space lesions. MRI findings may be atypical—especially in the early acute phase. Although the exact pathophysiological mechanisms leading to dorsal migration remain debatable, specific anatomical and biomechanical factors, including the location of the PLL tear and the anatomical spinal level, seem to play a critical role in facilitating this atypical migration trajectory. Neurological presentation is acute and may be severe, including the evolution of CES. The full endoscopic interlaminar approach is a viable option, presenting distinct perioperative benefits. The recovery usually requires substantial time, however prompt surgical decompression, particularly in younger patients, can lead to a faster clinical recovery.

## Figures and Tables

**Table 1:** MRI presentation of the case and the differential diagnosis of the most common dorsal intraspinal lesions. Reference of signal intensity is the myelon (according to general practice regarding extramedullary lesions) [12,17,18,19].

Pathology	T1-Weighted Signal	T2-Weighted Signal	Contrast Enhancement Pattern	Key Distinguishing Features
**Presented Case (Dorsal Disc Sequestration)**	Isointense	Isointense	None	-
**Classic Disc Sequestration**	Isointense	Hyperintense	Peripheral ring-enhancement	Inflammatory granulation tissue causes a rim enhancement
**Epidural Abscess**	Low to moderate	High	Homogeneous or peripheral	Elevated inflammatory markers (CRP/WBC); systemic infection symptoms.
**Epidural Hematoma**	Variable (depends on stage)	High (fluid-like)	Minimal to none (circular/rim enhancement in chronic stage)	History of trauma, rheumatologic diseases (ankylosing spondylitis; diffuse idiopathic skeletal hyperostosis), anticoagulation or sudden acute onset; changes signal intensity over time.
**Synovial Cyst**	Low	High	Minimal to none (thin peripheral rim)	Spherical, well-defined fluid-filled lumen; may extend in the epidural space in Baastrup’s disease (kissing spine syndrome) [20,21]
**Neurilemmoma (Schwannoma)**	Isointense	Hyperintense	Intense homogeneous	Well-circumscribed benign tumor; often follows the course of an exiting nerve root.
**Meningioma**	Isointense	Isointense	Intense homogeneous	Broad-based dural attachment (“dural tail sign”); common in thoracic spine
**Lipoma**	High (Hyperintense)	High (Hyperintense) Hypointense on fat-suppressed T2 sequence	None	Complete signal drop-out on fat-suppressed (STIR) sequences.
**Vertebral Metastasis**	Low	High	Variable/Infiltrative	Osteolytic/sclerotic destruction of the vertebral body or posterior arch; T2 “halo sign”
**Ligamentum flavum cyst**	Homogeneous very low signal intensity	High	Intense mural enhancement	No continuity with facet joint. In rare cases secondary hemorrhage; infection may occur, resulting in hyperintense T1 and hypointense T2 signals

## Data Availability

The original contributions presented in this study are included in the article/Supplementary Material. Further inquiries can be directed to the corresponding author.

## Supplementary Materials

The following supporting information can be downloaded at: https://www.mdpi.com/article/10.3390/reports9040361/s1, Video S1: Video presentation of the excision of a massive disc fragment.

## Abbreviations

The following abbreviations are used in this manuscript:

MRI	Magnetic Resonance Imaging
FESS	Full Endoscopic Spine Surgery
CES	Cauda Equina Syndrome
CESI	Cauda Equina Syndrome Incomplete
PEMDF	Posterior Epidural Migration of Disc Fragment
Hct	Hematocrit
V	Volume
RBC	Red Blood Cells
